# Enabling and disabling behaviors in the social environment are associated with physical Activity of older people in the Netherlands

**DOI:** 10.1186/s12889-019-6670-z

**Published:** 2019-04-01

**Authors:** Anna P. Nieboer, Jane M. Cramm

**Affiliations:** Department of Social Medical Sciences, Erasmus School of Health Policy and Management, Erasmus University Rotterdam, P.O. Box 1738, 3000 DR Rotterdam, The Netherlands

**Keywords:** Physical activity, Active ageing, Social influence, Older people, Health behaviors

## Abstract

**Background:**

Although research clearly shows that physical activity has significant health benefits and contributes to the prevention of chronic disease onset, the vast majority of the world’s population is insufficiently physically active, and the prevalence of insufficient physical activity is greatest in the population of older adults. The social environment may play an important role in shaping health behaviors, we however, lack knowledge regarding the exact influence of the social environment on older persons’ physical activity levels. This research therefore aims to identify the relationships of physical activity to enabling and disabling behaviors in the social environment among older people in the Netherlands.

**Methods:**

Participants were randomly sampled from the Rotterdam municipality register and stratified by age group (70–74, 75–79, 80–84, and ≥ 85 years) and neighborhood (district). Of 2798 respondents, 1280 (46%) returned filled-in questionnaires. The Perceived Social Influence on Health Behavior (PSI-HB) instrument was used to assess the degree to which individuals’ health behavior is influenced by those around them. Respondents were additionally asked about enabling and disabling behaviors in their social environments and how many days per week they were physically active. Physical activity scores ranged from 0 (not being physically active for 30 min a day at all during the week) to 7 (being physically active every day of the week). Respondents with a score of ≥5 were considered to be physically active and those with a score of < 5 as physically inactive.

**Results:**

Results revealed that increasing age significantly contributed to physical *in*activity within this older population. Lower educational level significantly decreased the odds of physical activity. After controlling for background characteristics results show enabling behaviors and utilitarian social influence significantly increased the odds of physical activity while disabling behaviour of the social environment contributed to physical *in*activity. No significant associations were found with perceived social influence aspects value-expressive influence and informational influence.

**Conclusion:**

Actual enabling and disabling behaviors of actors in older people’s social environments seem relevant for their physical activity levels, in positive and negative ways. In promoting active aging, consideration of the role of the social environment and ensuring that it is supportive of older people’s physical activity are important.

**Electronic supplementary material:**

The online version of this article (10.1186/s12889-019-6670-z) contains supplementary material, which is available to authorized users.

## Background

Physical inactivity has been identified as a leading risk factor for global mortality [1–3]. Furthermore, insufficient physical activity is a key risk factor for many chronic conditions, such as cardiovascular diseases, cancer, diabetes [3], depression [4], and anxiety [5, 6]. Even a slight increase in activity may reduce a person’s risk of death [7], and it elevates mood and energizes individuals [8, 9]. In addition, high levels of physical activity have been shown to improve overall well-being [10–12].

Although research clearly shows that physical activity has significant health benefits and contributes to the prevention of chronic disease onset, more than 80% of the world’s population is insufficiently physically active, and the prevalence of insufficient physical activity is greatest in the population of older adults [2]. In a time of aging populations, the investigation of ways in which to encourage healthy and active aging is particularly vital. The promotion of active aging through physical activity has the potential to slow the otherwise ever-growing burden on national economies and health care systems worldwide, and, more importantly, to ensure that older people are able to enjoy their lives to the best of their capacities.

Thus, the investigation of ways in which to increase physical activity among older adults is essential. Even in countries with policies to address insufficient physical activity in operation, a large proportion of the population is physically inactive. The social environment may play an important role in shaping health behaviors, in addition to the most important enablers/disablers of physical activity among community-dwelling older adults: advanced age, income, and educational level [13]. In a systematic review of impediments to and enablers of physical activity, Siddiqi, Tiro, & Kerem Shuval [14] found that a person’s social environment may be an important enabler. In another study, the social environment was also identified as a potentially important enabler of physical activity among people with chronic lung conditions [15]. Despite this research, however, we lack knowledge regarding the exact influence of the social environment on older persons’ physical activity levels.

### Perceived social influence on health behaviors

The effect of social influence on health behavior has been recognized to be of a multidimensional nature. Bearden and colleagues [16] identified three dimensions of social influence: *utilitarian influence*, *value-expressive influence*, and *informational influence* [17; p. 2]. Under utilitarian influence, behavior occurs when a person is rewarded for it by significant others (e.g., “I rarely engage in health behaviors until I am sure my friends approve of them,” “I like to know what health behaviors make good impressions on others”; [16–18]. Under value-expressive influence, behavior is influenced by a reference group to which a person wants to belong. Value-expressive influence involves identification processes (e.g., “If I want to be like someone, I often try to make the same healthy choices that they do”; “I often identify with other people by making the same healthy choices that they do” [16–18]. Informational influence occurs when a person looks for information from others to form his or her reality, which involves internalization processes (e.g., “I often consult other people to help choose the best alternative available for a health behavior,” “I frequently gather information from friends and family before I engage in a health behavior” [16–19]. Consideration of these dimensions of social influence may aid understanding of the roles of social networks and social influence processes on health behaviors such as physical activity, which can be positive and negative [17]. The relationship is indirect in nature, and does not involve actual behavior of people comprising the environment; (a change in) behavior occurs when a person perceives that the social environment will approve such behavior and reward him/her for it. Conversely, a given behavior or lack of support by significant others can present a significant barrier to behavioral change. Significant others may thus encourage or discourage health behaviors. As explained by Bandura’s [20, 21] social learning theory, a change in behavior is a cognitive process that takes place in a social context. A behavior that is often rewarded in the immediate environment will likely persist, whereas regular punishment of an unhealthy behavior will likely lead a person to desist.

### Actual enabling and disabling behaviors of actors in the social environment

The *actual behavior* of people comprising the social environment has also been identified as important for health behaviors [22]. People in one’s social environment, for example, might tell one to be careful and slow down because of a chronic condition (disabling behavior for physical activity), whereas they should instead advise one to be more active (enabling behavior for physical activity). In turn, behavior that extends beyond advising people on being physically active, such as joining them in a physical activity (e.g., taking a walk together), may be an important enabler of physical activity in particular [22].

### Study aim

Although the effect of social influence and behaviors within the social environment have been found to be associated with several health behaviors, such as smoking [23], smoking intention [24], and fruit and vegetable consumption [25], relationships between physical activity and enabling and disabling behaviors in the social environment among older people remain poorly understood and understudied. Therefore, this research aimed to identify such relationships among older people in the Netherlands. Utilitarian, value-expressive, and informational influence were investigated with consideration of older people’s lifestyles in general in relation to their levels of physical activity. In addition, we investigated enabling and disabling behaviors in the social environment related to physical activity, in relation to older people’s actual physical activity (e.g., “Have people who are important to you taken you for a walk, swim, or bike ride lately?”; “Have people who are important to you told you that it is important to stay physically active lately?”).

## Methods

Participants were randomly sampled and stratified by age group (70–74, 75–79, 80–84, and ≥ 85 years) and neighborhood (district). They were identified using the Rotterdam municipality register. The number of participants per neighborhood was proportionally weighted according to the population ratio of the district. Only one person per address was allowed to participate. Participants were sent a written questionnaire with an invitation to participate in the study and a self-addressed envelope. Two reminders were sent by mail in cases of non-response. In total, 2890 people were approached. Sixty-seven respondents were excluded, as they resided in nursing homes or were hospitalized. Another 25 respondents could not participate due to serious medical issues (i.e., dementia) or death. This information was obtained from the respondent’s proxy. Of the remaining 2798 respondents, 1280 returned filled-in questionnaires, achieving a response rate of 46%.

The research proposal has been reviewed by the medical ethics committee of Erasmus Medical Centre (study protocol number MEC-2011-197). The committee decided that the rules laid down in the Medical Research Involving Human Subjects Act did not apply. Informed consent to participate in the study was obtained from all participants.

### Measures

#### Perceived social influence on health behavior instrument

The Perceived Social Influence on Health Behavior (PSI-HB) instrument was designed to assess the degree to which individuals’ health behavior is influenced by those around them [17]. The PSI-HB is a 10-item measure of the perceived roles of others in the health behavior decisions of individuals. Responses in three subscales (utilitarian influence [4 items], value-expressive influence [3 items], and informational influence [3 items]) are structured by a 4-point Likert-type format (strongly disagree (1), disagree (2), agree (3), strongly agree (4)) (see Additional files 1). Mean scale scores were calculated on each subscale. Alpha values reflecting the internal consistency of the subscales ranged from 0.85 to 0.88. The original English-language PSI-HB items were translated into Dutch by a professional translator.

#### Enabling and disabling behaviors

Respondents were asked 4 questions about enabling and disabling behaviors in their social environments. Responses are structured by a four-point scale (no, now and then, regularly, often; see Additional files 2) [22]. Total scores were calculated by summing item responses (theoretical range, 2–8). Higher scores on the enabling subscale indicate promotion of physical activity by the social environment and higher scores on the disabling subscale indicate that the social environment tends to create physical inactivity among respondents.

#### Physical Activity

Respondents were asked about their physical activity by asking how many days per week (referring to a ‘normal’ week in recent months) they were physically active for at least 30 min each day. The following activities were included: actively commuting (walking, cycling), physical activity at work, household activities, leisure time activities (sports, walking, gardening, cycling). Scores ranged from 0 (not being physically active for 30 min a day at all during the week) to 7 (being physically active every day of the week). Respondents with a score of ≥5 were considered to be physically active and those with a score of < 5 as physically inactive [26–30]. This instrument has been proven to be reliable and valid to measure physical activity [27, 28].

#### Demographics

The questionnaire additionally asked respondents for information on their age, gender, educational level, and marital status. Patients’ educational levels were grouped into low educational level (no school or primary education only), medium educational level (preparatory secondary vocational education), and high educational level (senior general secondary education, university preparatory education). Marital status was dichotomized into married (those who were married or living together) and those who were unmarried (single, widowed or divorced).

### Analysis

We employed descriptive statistics and used logistic regression analysis to assess the relationships of physical activity with perceived social influence on health behavior, enabling and disabling behaviors in the social environment, and individual characteristics (age, gender, educational level, and marital status). Results were considered statistically significant when two-sided *p* values were ≤ 0.05 (SPSS ver. 22; IBM Corporation, Armonk, NY, USA).

## Results

Table 1 displays descriptive statistics for all independent variables and physical activity. Of the 1280 respondents, 42% were men. Their average age was 79.03 (range, 70–99; SD, 6.21) years, 41% of the respondents were married, 32% had low educational levels, 54% medium and 14% higher educational levels. About half (52%) of the respondents met the norm of leading a physically active life (at least 30 min of physical activity on at least 5 days a week).

**Table 1:** Participant Characteristics

Characteristic	*n*	Percentage	Mean ± standard deviation (range)
Age (years)	1280		79.03 ± 6.21 (70–99)
Gender (male)	1280	42%	
Marital status (married)	1255	41%	
Educational level	1280		
Low		32%	
Medium		54%	
High		14%	
Physical activity (days per week)	1220		4.15 ± 2.43 (0–7)
0 days per week 1 day per week 2 days per week 3 days per week 4 days per week 5 days per week 6 days per week 7 days per week	142 88 124 127 103 188 133 315	12% 7% 10% 10% 9% 15% 11% 26%	
Utilitarian influence	1199		7.72 ± 3.00 (4–16)
Value-expressive influence	1207		5.29 ± 2.19 (3–12)
Informational influence	1200		5.64 ± 2.27 (3–12)
Enabling behavior	1205		4.38 ± 1.42 (2–8)
Disabling behavior	1199		4.57 ± 1.76 (2–8)

Table 2 presents the results of the logistic regression analysis. Looking at background characteristics this analysis revealed that increasing age significantly contributed to physical inactivity within this older population. Lower educational level significantly decreased the odds of physical activity (compared to being higher educated). The regression analysis revealed no significant relationships between physical activity, gender and marital status (Table 2). After controlling for background characteristics results show enabling behaviors and utilitarian social influence significantly increased the odds of being physically active while disabling behaviour of the social environment contributed to physical inactivity. No significant associations were found with perceived social influence aspects value-expressive influence and informational influence.

**Table 2:** Results of Logistic Regression Analysis of Being Physically Active and Individual Characteristics, Perceived Social Influence, and Enabling and Disabling Behaviors in the Social Environment (*n* = 1280)

Model	B	SE	Wald	OR (95% CI)	*P*
*Individual characteristics*					
Age (years)	−0.04	0.01	10.66	0.964 (0.942–0.985)	< 0.001
Gender (female)	−0.07	0.15	0.25	0.929 (0.694–1.244)	0.620
Marital status (married)^a^	−0.06	0.15	0.15	0.945 (0.709–1.261)	0.702
Educational level (low)^b^	−0.61	0.21	8.20	0.542 (0.356–0.824)	0.004
Educational level (medium)^b^	−0.28	0.19	2.13	0.753 (0.515–1.102)	0.114
*Perceived social influence*					
Utilitarian influence	−0.07	0.03	4.11	0.936 (0.878–0.998)	0.043
Value-expressive influence	0.04	0.05	0.75	1.044 (0.947–1.151)	0.388
Informational influence	−0.04	0.04	1.00	0.962 (0.892–1.038)	0.318
*Enabling and disabling behaviors*					
Enabling behavior	0.27	0.05	28.69	1.303 (1.183–1.436)	< 0.001
Disabling behavior	−0.09	0.04	5.05	0.911 (0.840–0.988)	0.025
Constant	3.19	0.92	12.00	24.373	

Listwise deletion of missing cases resulted in the inclusion of 1069 cases in the regression analysis. B = unstandardized regression coefficient. ^a^Reference group unmarried (single, widowed or divorces). ^b^Reference group higher educational level

## Discussion

The improvement of physical activity levels through the social environment among older people remains poorly understood. Therefore, this research aimed to identify the relationships of physical activity with enabling and disabling behaviors in the social environment among older people in the Netherlands. The results showed that *actual* enabling and disabling behaviors in the social environment were especially important for physical activity among older people. The logistic regression analysis showed that enabling behavior in the social environment increased the odds for older people’s physical activity, whereas disabling behavior decreased these odds. These behaviors can be informational (by saying walking is good for you), in the form of advisements (telling another person they should exercise more) and/or actual behaviors of the social environment (e.g. another person in your social network who walks or exercises with you). These findings suggest that interventions seeking to improve older people’s engagement in physical activity should specifically consider family members and friends as important sources to promote active aging through actual enabling behaviors, such as those supporting leisure-time physical activity [31]. “Buddy”-style interventions, in which people are encouraged to exercise with significant others, have been successful in the general population [32] and among older people [33]. Not only the encouragement of healthy behavior, but also the setting of a good example regarding physical activity level by people in older people’s immediate surroundings is expected to contribute to active aging. According to Bandura [20, 34], new behaviors can be acquired by observing and imitating others (so-called vicarious reinforcement). Although these behavioral theories were based on data from general populations, the creation of supportive social and physical environments for physical activity also has been found to improve physical activity in older populations. Programs that are responsive to the dynamic interaction of the individual with the environment seem to be beneficial in this regard [35].

In terms of the *perceived influence* of the social environment, we found utilitarian influence decreased the odds of physical activity among older people. Those who are more in agreement with the items ‘I rarely engage in health behaviors until I am sure my friends approve of them’, ‘It’s important that others agree with my health lifestyle [before I act]’, ‘When engaging in health behaviors, I generally do things that I think others will approve of’ and ‘I like to know what health behaviors make good impressions on others’ are less physically active compared to those who are less in agreement with these items. Researchers have previously proposed that individuals and behaviors can be subject to normative control [36], suggesting that views of social influence differ. Holt and colleagues [17] found that men are more susceptible than women to social influence on health behaviors. They also found a negative association between utilitarian influence and physical activity, which they took to reflect the negative role of social networks or peer influences. Alternatively, people who are more susceptible to other people’s views, approval, or opinions may simply do less (in this case, be less physically active). Our questionnaire did not enable assessment of this possibility, as it did not distinguish between positive and negative social influence (favorable or unfavorable to physical activity) or indicate whether the respondent was susceptible to such behavior. More research is needed to unravel the relationships between the actual and perceived influences of the social environment on physical activity in older populations and possible differences between men and women or other confounders such as mental and physical health.

Results of this study also show that 52% of Rotterdam residents aged ≥70 years were physically active (at least 30 min per day, at least 5 days a week). This finding is in line with data from the general population of older Dutch people in the same time period (2010–2013), which indicate that 57.9% of people aged 65–74 years and 49.4% of those aged ≥75 years meet the norm of physical activity [30]. About half of the older population can thus be considered to be inactive, which may be harmful to health and warrants improvement.

This research has several limitations. An important limitation is the cross-sectional nature of the data, which enabled the identification of associations, but not causality. The results of this study show that the concepts of enabling and disabling support provide insight into behaviors in the environment and the health behaviors of older people. The next step is to study these concepts from a longitudinal perspective to aid understanding of the underlying processes. Cross-sectional data may underestimate the influence of the environment, as health-damaging behaviors of older people may evoke enabling support and healthy behaviors may restrain the provision of such support. In addition, we investigated a population of older people from a single municipality in the Netherlands; it would be interesting to repeat the study in other settings, given that the role of the social environment is known to differ across cultures. Another limitation is that the Perceived Social Influence on Health Behavior (PSI-HB) instrument has not been validated among older people in the Netherlands yet. While we have no indication that the perceived roles of others in the health behavior decisions of individuals would be different in the Netherlands than America (where the instrument was developed and validated) we do not know this for sure. Furthermore, we included only a few co-variates in the multivariate analysis and self-reported measures only. We did not assess actual behavior or physical activity (e.g. by constantly monitoring a respondent’s physical activity). Use of other instruments may lead to different findings. Finally, although our 46% response rate is higher compared to other studies where respondents also received a questionnaire by mail [37, 38], it is lower compared to studies where respondents were visited by interviewers in their homes [39] which may have resulted in non-response bias. Poorer health and as a result lower levels of physical activity, could be higher among non-responders leading to a potential non-response bias. However, activity levels in our sample were comparable to the general population of older Dutch people as reported above. Furthermore, our main interest concerns the relationship between enabling and disabling behaviors in the social environment with physical activity of older people in the Netherlands. Given that we have enough variation in our sample regarding background characteristics, physical activity, enabling and disabling behaviors in the social environment we are confident that this has not affected our study findings. To further investigate the possibility of response bias regarding background characteristics, we also compared the characteristics of the study sample (*n* = 1280) and the original sample (*n* = 2798). We found no difference in age, but a significant difference in gender (42% male participants vs. 38% men in the original sample), which may indicate selective non-response. The percentage of lower educated community-dwelling older people is similar to other studies among the same age group of community-dwelling older people in Rotterdam [40].

Strengths of the study are a relatively large study sample of community-dwelling older people residing in Rotterdam and using both perceived social influence as well as actual behavior of the social environment in the same study.

## Conclusions

Based on the results of this study, we can conclude that in addition to the known predictors (age and educational level), actual enabling and disabling behaviors of actors in older people’s social environments seem relevant for their physical activity levels, in positive and negative ways. These findings are important in a time of aging populations and the increasing importance of active aging. In promoting active aging, consideration of the role of the social environment and ensuring that it is supportive of older people’s physical activity are important.

## Additional files

Additional file 1: Instrument to assess perceived social influence on health behaviors. (DOCX 12 kb)

Additional file 2: Assessment of Enabling and Disabling Behavior in the Social Environment, Developed by Nieboer (1992). (DOCX 12 kb)

## Abbreviation

PSI-HB: Perceived Social Influence on Health Behavior
SQUASH: Short Questionnaire to Assess Health-Enhancing Physical Activity
