# Supplementary material for: Enabling and disabling behaviors in the social environment are associated with physical Activity of older people in the Netherlands
Source: BMC Public Health. 2019 Apr 1;19:361. doi: 10.1186/s12889-019-6670-z (PMC6444431; doi:10.1186/s12889-019-6670-z)
Supplement: Supplementary file 1 — Instrument to assess perceived social influence on health behaviors. (DOCX 12 kb) [file 12889_2019_6670_MOESM1_ESM.docx]

**Additional file 1. Instrument to assess perceived social influence on health behaviors**

Developed by Holt and colleagues (2010). Published in final edited form as: J Health Psychol . 2010 November ; 15(8): 1225–1235.

For the next few items, I am going to refer to ‘health behaviors’. What I mean by ‘health behaviors’ is anything that you do that impacts your health, like going to the doctor, your eating habits, or physical activity. Please respond to the following statements with Strongly Agree, Agree, Disagree, or Strongly Disagree.

*Utilitarian influence*

I rarely engage in health behaviors until I am sure my friends approve of them.

It's important that others agree with my health lifestyle [before I act].

When engaging in health behaviors, I generally do things that I think others will approve of.

I like to know what health behaviors make good impressions on others.

*Value-expressive influence*

I achieve a sense of belonging by making the same healthy choices that others do.

If I want to be like someone. I often try to make the same healthy choices that they do.

I often identify with other people by making the same healthy choices that they do.

*Informational influence*

If I have little experience with a health behavior, I often ask my friends about it.

I often consult other people to help choose the best alternative available for a health behavior.

I frequently gather information from friends and family before I engage in a health behavior
