# Supplementary material for: Enabling and disabling behaviors in the social environment are associated with physical Activity of older people in the Netherlands
Source: BMC Public Health. 2019 Apr 1;19:361. doi: 10.1186/s12889-019-6670-z (PMC6444431; doi:10.1186/s12889-019-6670-z)
Supplement: Supplementary file 2 — Assessment of Enabling and Disabling Behavior in the Social Environment, Developed by Nieboer (1992). (DOCX 12 kb) [file 12889_2019_6670_MOESM2_ESM.docx]

**Additional file 2. Assessment of Enabling and Disabling Behavior in the Social Environment, Developed by Nieboer (1992)**

The following questions concern the people who are in close contact with you and who matter the most to you. For example, your partner, children, family members, friends, close acquaintances and neighbors. The questions are about what these people have done and told you lately when it comes to your physical activity. They are not about what your own actions are, but what people who matter to you say or do. Could you please indicate which answer is most applicable to your situation?

***Lately, have people who matter to you..........***

1. *...... told you it is important to stay physically active?*  No

Now and then

Regularly

Often

2. *...... gone walking, swimming or biking together with you?*  No

Now and then

Regularly

Often

3. *...... told you to be careful because they are afraid you might fall?*  No

Now and then

Regularly

Often

4. *...... told you to slow down?* No

Now and then

Regularly

Often
